# SMRT sequencing of the full-length transcriptome of *Odontotermes formosanus* (Shiraki) under *Serratia marcescens* treatment

**DOI:** 10.1038/s41598-020-73075-3

**Published:** 2020-09-28

**Authors:** Kai Feng, Xiaoyu Lu, Jian Luo, Fang Tang

**Affiliations:** 1 Co-Innovation Center for Sustainable Forestry in Southern China, Nanjing Forestry University, Nanjing, 210037 China; 2 College of Forestry, Nanjing Forestry University, Nanjing, 210037 China

**Keywords:** Entomology, RNA sequencing

## Abstract

*Odontotermes formosanus* (Shiraki) is an important pest in the world. *Serratia marcescens* have a high lethal effect on *O. formosanus,* but the specific insecticidal mechanisms of *S. marcescens* on *O. formosanus* are unclear, and the immune responses of *O. formosanus* to *S. marcescens* have not been clarified. At present, genetic database resources of *O. formosanus* are extremely scarce. Therefore, using *O. formosanus* workers infected by *S. marcescens* and the control as experimental materials, a full-length transcriptome was sequenced using the PacBio Sequel sequencing platform. A total of 10,364 isoforms were obtained as the final transcriptome. The unigenes were further annotated with the Nr, Swiss-Prot, EuKaryotic Orthologous Groups (KOG), Gene Ontology (GO) and Kyoto Encyclopedia of Genes and Genomes (KEGG) Ortholog public databases. In a comparison between the control group and a *Serratia marcescens-*infected group, a total of 259 differentially expressed genes (DEGs) were identified, including 132 upregulated and 127 downregulated genes. Pathway enrichment analysis indicated that the expression of the mitogen-activated protein kinase (MAPK) pathway, oxidative stress genes and the AMP-activated protein kinase (AMPK) pathway in *O. formosanus* may be associated with *S. marcescens* treatment. This research intensively studied *O. formosanus* at the high-throughput full-length transcriptome level, laying a foundation for further development of molecular markers and mining of target genes in this species and thereby promoting the biological control of *O. formosanus.* Furthermore, these results will be helpful to clarify the action mechanisms of *S. marcescens* on *O. formosanus,* and also explore the relationship between *O. formosanus* and *S. marcescens*. In addition, this study will identify the immune response of *O. formosanus* to *S. marcescens*, which will provide a theoretical foundation for the development of new immunosuppressants for *O. formosanus*.

## Introduction

Termites are known for their destructive potential, and they harm buildings, agricultural and forestry crops, the earth dams of reservoirs, cable bridges, traffic facilities, and more, and they often eat noncellulose materials^1–3^. Termites are widely distributed and cause billions of property losses every year. Because of the complex feeding habits of termites, they can cause more or less severe harm to human buildings, vehicles, crops, and so on. More serious effects can include the collapse of houses and result in threats to human life, causing irreparable damage. In 1992, the number of known termite species in China had reached 476, and the termite damage to the south of the Yangtze River was the most serious. At that time, China’s annual damage due to termites had reached more than 2 billion yuan. The economic loss caused by termite damage in the United States in 1998 was approximately $5 billion^4^.

*Odontotermes formosanus* (Shiraki) is a derived type of termite that remains underground most of the time. The workers of this species mainly damage bark and roots, and these termites prefer humid but not wet conditions^5^. Donkeys, mountain frogs, bats, various ants, spiders, pangolins, and other animals are their natural enemies^6^. *O. formosanus* is the main pest in reservoir and river dams, often causing dam leakage, collapse and breach^7^. Li et al. found that the presence of *O. formosanus* nest has a great impact on the overall and local stability of the embankment. The existence of termite nest reduces the maximum safety factor of the downstream slope by 17%^8^. Because the nests of *O. formosanus* are very well concealed, the damage caused by this species is not easily detected. When it is detected, a great deal of damage is already present. In addition, *O. formosanus* is a generalist and can damage more than 100 kinds of plants, including eucalyptus, cedar, magnolia, and maple^9^. Kirton et al.^10^ found the phenomenon of ring-barking and root debarking of saplings in the Malaysian peninsula of Southeast Asia. At the same time, many termite species of termite genera, including *Schedorhinotermes*, *Odontotermes*, *Procapritermes*, *Pericapritermes* and *Nasutitermes*, were found on or near the main roots of the saplings, among which the most common was *Odontotermes*.

Currently, increasing attention is being paid to the prevention and control of termites in China. Large amounts of manpower, material resources and financial resources have been invested in termite prevention and control to minimize termite-related losses to human beings. At present, imidacloprid, fipronil, chlorpyrifos, fenvalerate and bifenthrin are commonly used termite control agents. Although chemical control can achieve certain results, there will be pesticide residues, which will cause serious harm to the ecological environment. At the same time, due to the increase in human environmental awareness, green pesticides are becoming increasingly popular. The research and development of new termite pesticides is currently a hot topic. Notably, in the field of biological control, *Serratia marcescens* can effectively control some pests and pathogens^11,12^. However, *Serratia marcescens* strains from different sources have very different characteristics. Previously, we isolated a strain of *S. marcescens* from *O. formosanus*. After separation and purification, a pure strain was obtained^13^. Then, the bioassay showed that *S. marcescens* have a high lethal effect on *O. formosanus*^14^. At the same time, we found that the insecticidal effect of *Metarhizium anisopliae* and *S. marcescens* was better than *M. anisopliae* or *S. marcescens* individually^14^. In addition, we reported that the main components that affected *O. formosanus* were in a supernatant and that the insecticidal protease in the supernatant resulted in the death of *O. formosanus*^15^.These results showed that *S. marcescens* had good insecticidal effect on *O. formosanus*, but the action mechanisms of *S. marcescens* on *O. formosanus* need to be further clarified. Guo et al. demonstrated that MAPK signaling pathway is a general switch, which can transregulate the differential expression of genes in the midgut of *Plutella xylostella*, so as to resist the toxic effect of *Bacillus thuringiensis* (Bt)^16^. So, we speculate that the signal transduction and energy metabolism of *O. formosanus* will be affected in varying degrees, and the oxidative stress will be affected when infected by *S. marcescens*.

In recent years, third-generation high-throughput sequencing technology characterized by single-molecule real-time (SMRT) sequencing has been successfully applied to functional genome research in yeast^17^, rabbit^18^, sorghum^19^, corn^20^, wheat^21^ and cotton^22^. Compared with second-generation sequencing technology, third-generation sequencing technology not only has the advantages of large data sets, long sequence reads and full-length gene transcripts but also does not require sequence splicing and assembly, which greatly improves the accuracy of gene functional annotation^23^. However, this technology has not previously been used in termites. Therefore, this study used *O. formosanus* as an experimental material to perform full-length transcriptome sequencing to explore the key metabolic pathways, signaling pathways and genes affected by *S. marcescens*, and analysis of *O. formosanus* infected with *S. marcescens* using the third-generation high-throughput sequencing technology to clarify the action mechanisms of *S. marcescens* on *O. formosanus*. Furthermore, these data also explored the relationship between *O. formosanus* and *S. marcescens*, which will provide theoretical basis for biological control of *O. formosanus* by using *S. marcescens*. In addition, this study will identify the immune response of *O. formosanus* to *S. marcescens*, which will provide a theoretical foundation for the development of new immunosuppressants for *O. formosanus*.

## Materials and methods

### Insects and pathogenic bacteria

*Odontotermes formosanus* was collected from JuRong (Jiangsu Province, China), and the nests were harvested. Then, the nests were placed in an insect incubator under dark conditions (L:D = 0:24) and 75 ± 1% relative humidity at 27 ± 1 °C. The *S. marcescens* strain SM1 was previously isolated from infected and dead *O. formosanus* and stored in glycerin at − 80 °C.

### Sample processing

The isolated *Serratia marcescens* was transferred to a bacterial basal medium and cultured in the dark at 27 °C for 24 h. After streaking, a single colony was removed, placed in a 250 mL Erlenmeyer flask containing 50 mL of seed culture medium, and cultured for 12 h at 30 °C in a constant temperature shaker at 200 r/min . Then, 70 mL of the seed solution was added to 250 mL of the fermentation medium and cultured in a shaking incubator at a temperature of 30 °C for 36 h at a rate of 200 r/min. The cultured fermentation medium was used in subsequent experiments. Then, filter paper was placed on the bottom of a 9 cm petri dish and wetted with water. Twenty mature worker termites with good vitality were placed into the petri dish, and ten petri dishes were set up. In the *S. marcescens*-infected experimental group (SMT), 0.12 μL of *S. marcescens* solution (culture fermentation medium) was collected and dropped on pronotum of termites. For the control group (CK), 0.12 μL sterile liquid (fermentation medium without *S. marcescens*) was placed in the same locations on the termites. After 20 h of treatment, the ten termites were collected and stored in liquid nitrogen for subsequent experiments in the CK and the SMT respectively. The CK and the SMT were repeated three times independently.

### RNA sample preparation

After the tissue was taken out, it was frozen in liquid nitrogen, then stored in − 80 °C. Total RNA was extracted by Trizol method. Agarose gel electrophoresis was used to analyze the degree of degradation and whether there was contamination in RNA solution. Then, RNA purity was checked using a NanoPhotometer spectrophotometer (IMPLEN, CA, USA). Next, RNA concentration was measured using a Qubit RNA Assay Kit in a Qubit 3.0 Fluorometer (Life Technologies, CA, USA). RNA integrity was assessed using the RNA Nano 6000 Assay Kit of the Agilent Bioanalyzer 2100 system^24,25^.

### Library preparation and SMRT sequencing

A total of 1 μg RNA per sample was used as input material for RNA sample preparation. Sequencing libraries were generated using the NEBNext Ultra RNA Library Prep Kit for Illumina (NEB, USA) following the manufacturer’s recommendations, and index codes were added to attribute the sequences to each sample^24–26^. In short, mRNA was purified from the total RNA using poly-T oligo-attached magnetic beads. Fragmentation was carried out using divalent cations under elevated temperature in NEBNext First Strand Synthesis Reaction Buffer (5×)^26^. The first strand of cDNA was synthesized by random hexamer primer and M-MuLV Reverse Transcriptase (RNaseH-). The second strand of cDNA was synthesized by DNA polymerase I and RNase H. The rest overhangs were transformed into blunt ends via exonuclease/polymerase activities. After adenylation of the 3′ ends of the DNA fragments, NEBNext Adaptor with hairpin loop structure was connected to prepare for hybridization. In order to select the proper length of cDNA fragment, the library fragment was purified by AMPure XP system (Beckman Coulter, Beverly, USA). Then, 3 μL USER Enzyme (NEB, USA) was incubated with the size-selected, adaptor-ligated cDNA at 37 °C for 15 min followed by 5 min at 95 °C. PCR was then performed with Phusion High-Fidelity DNA polymerase, Universal PCR primers and Index (X) Primer. Finally, the products were purified (AMPure XP system), and library quality was assessed on the Agilent Bioanalyzer 2100 System^25,26^. The library preparations were sequenced on the PacBio Sequel platform.

### Sequel data output and quality control

The PacBio sequence sequencing platform is based on single-molecule real-time (SMRT) sequencing technology. We used SMRT Link to preprocess and filter the original sequencing output data. The main parameters were Minimum Subread Length = 50, Maximum Subread Length = 15,000, Minimum Number of Passes = 3, Minimum Predicted Accuracy = 0.8, Minimum Read Score = 0.65, and Minimum Polished Isoform Accuracy 0.99; the remaining parameters were set to the default values. For ISO SEQ sequencing, the data preprocessing and filtering of SMRT Link mainly includes the following steps: the single-molecule polymerase reads are split to obtain subreads, and the subreads obtained from the same polymer reads are self-corrected and combined to form a circular consensus sequence (CCS). The CCSs were classified by detecting the presence of chimeric sequences, sequencing primers and 3′ poly-A sequences. CCSs with sequencing primer sequences, 3′ terminal poly-A sequences and no chimeric sequences were considered full-length nonchimeric (FLNC) sequences. The linker, primer and poly-A sequences corresponding to the FLNC sequences were removed, and all sequences were forward sequences. Then, the iterative clustering and error correction (ICE) tool of the SMRT Link software cluster module was used to cluster and correct multiple FLNC sequences of the same isoform to obtain the redundant isoform sequence. The non-FLNC sequences removed during the generation of the FLNC sequences were used to further correct the isoform sequence to enhance the sequence quality (cluster/polish) to obtain the polished isoform sequence. The polished isoforms were subjected to secondary sequence clustering by cd-hit-est software to further remove sequence redundancy. The sequences obtained in this step were the final non-redundant isoform set and were used for all subsequent analyses.

### Functional annotation of transcripts

The obtained non-redundant transcript sequences were mapped to five databases to obtain transcript annotation information.

The NCBI nonredundant protein sequences (Nr) include the protein-coding sequence of GenBank gene, Protein Data Bank (PDB) protein database, Swiss-Prot protein sequence and Protein Information Resource (PIR) and the protein sequence of a database such as Protein Research Foundation (PRF). Gene Ontology (GO) is a set of internationally standardized classification systems for gene function descriptions. GO is divided into three ontologies: biological process (BP), molecular function (MF) and cellular component (CC). The basic unit of GO is the term^27^. EuKaryotic Orthologous Groups (KOG) is a gene orthology database for eukaryotes. KOG combines evolutionary relationships to classify homologous genes from different species into different ortholog clusters. Orthologous genes have the same function, so functional annotations can be directly transferred to other members of the same KOG cluster^28^. Kyoto Encyclopedia of Genes and Genomes (KEGG) includes a systematic analysis of the metabolic pathways of gene products and compounds in cells and a database of the functions of these gene products. It integrates data about genomes, chemical molecules, and biochemical systems. The KO (KEGG Ortholog) system links the various KEGG annotation systems, and KEGG has established a complete KO annotation system to perform functional annotation of the genomes or transcriptomes of newly sequenced species^29^. The Swiss-Prot Protein Sequence Database (Swiss-Prot) contains carefully examined and accurately annotated protein sequences from the EMBL nucleic acid sequence database^27^.

### Digital gene expression library preparation and analysis

Using RSEM, the comparison results of Bowtie2 were counted, the number of reads from each sample compared to each transcript was obtained, and the fragments per kilobase per million bases (FPKM) was calculated. Paired-end reads from the same fragment were counted as one fragment, and then, the gene and transcript expression levels were obtained. A difference analysis was performed using the R language package DESeq2. To identify the differentially expressed genes (DEGs) between the SMT and CK libraries, the false discovery rate (FDR) method was used to determine the threshold of the P-value in multiple tests. The significance of the difference in gene expression was judged using a threshold of FDR < 0.05 and an absolute value of log_2_ fold change (FC, (condition 2/condition 1) for a gene) > 1 or log_2_^FC^ < − 1. Then, GO enrichment analysis and KEGG pathway enrichment analysis were used to further annotate the genes expressed at different levels among the samples.

## Results

### SMRT sequencing data output

Using the PacBio SMRT sequencing technology, 283,232 polymerase reads were generated (Table 1). With this technology, circular sequences are obtained from single molecules, and the effective inserts produced during the sequencing process are the polymerase reads. The sequence fragments remaining from the polymerase reads after removing the adaptor sequences are called subreads. After preprocessing, 20.06 Gb of subreads were obtained (Table 2). Circular Consensus Sequences (CCSs) are sequences with low error rates obtained by mutual correction of multiple sequencing results. A total of 218,147 CCSs were obtained (Table 3). A total of 172,632 FLNC with a mean length of 1418 bp were assembled from CCSs with an N50 length of 1478 bp.

**Table 1:** Statistics of polymer read data.

Sample	Library	Cell	Total bases (Gb)	Reads	Mean length (bp)	Read N50 (bp)
*O. formosanus*	Mix	Mix	21.02	283,232	74,207	158,725
Total	–	–	21.02	283,232	–	–

*Cells* the number of cells used for library construction, *Reads* the total number of read, *Mean length* average length of read, *N50* N50 length after filtration.

**Table 2:** Statistics of subread data.

Sample	Library	Cell	Total bases (Gb)	Reads	Mean length (bp)
*O. formosanus*	Mix	Mix	20.06	13,317,704	1,507
Total	–	–	20.06	13,317,704	–

*Cells* the number of cells used for library construction, *Total bases* the total number of subread, *Mean length* average length of subread.

**Table 3:** CCS statistics.

Library	Cell	Total bases (bp)	Reads	Mean read length (bp)	Mean read score	Passes
All_lib	All_cell	356,676,549	218,147	1635	0.99	53

*Cells* the number of cells used for library construction, *Total bases* the total number of read, *Mean read length* average length of read, *Mean Read Score* average quality of read, *Passes* number of cyclic sequencing.

### Transcript clustering analysis

Posttranscriptional modification of eukaryotic genes can produce a variety of different isoforms, and multiple FLNC sequences can be obtained from multiple sequencing of the same isoform. Using the ICE tools of the PacBio SMRT Link software to cluster the FLNC sequences and remove redundancy, a set of nonredundant isoform sequences can be obtained. Furthermore, the Quiver tool of the SMRT Link software was used to correct the isoform sequences obtained from the non-FLNC short sequences, and the obtained sequences were considered the polished isoforms. Then, a total of 10,364 polished isoform sequences with average length of 1493 bp were assembled from FLNC with an N50 length of 1648 bp. After the ICE clustering, 10,364 consensus isoforms were obtained, including 10,359 high-quality isoforms and five low-quality isoforms. The second sequence clustering was done by the software cd-hit-est to further remove the sequence redundancy. The sequence obtained in this step was the final non redundant isoform set and used for all subsequent analysis. Finally, a total of 9888 isoforms with average length of 1499 bp were assembled from polished isoform with an N50 length of 1659 bp, for further study.

### Alternative splicing analysis

A total of 24 alternative splicing events were identified, as shown in Supplementary Table S1. However, because no reference genome was available for SMRT transcriptome sequencing in *O. formosanus,* we were unable to determine the types of the alternative splicing events.

### Functional annotation of transcripts

**Figure 1:**
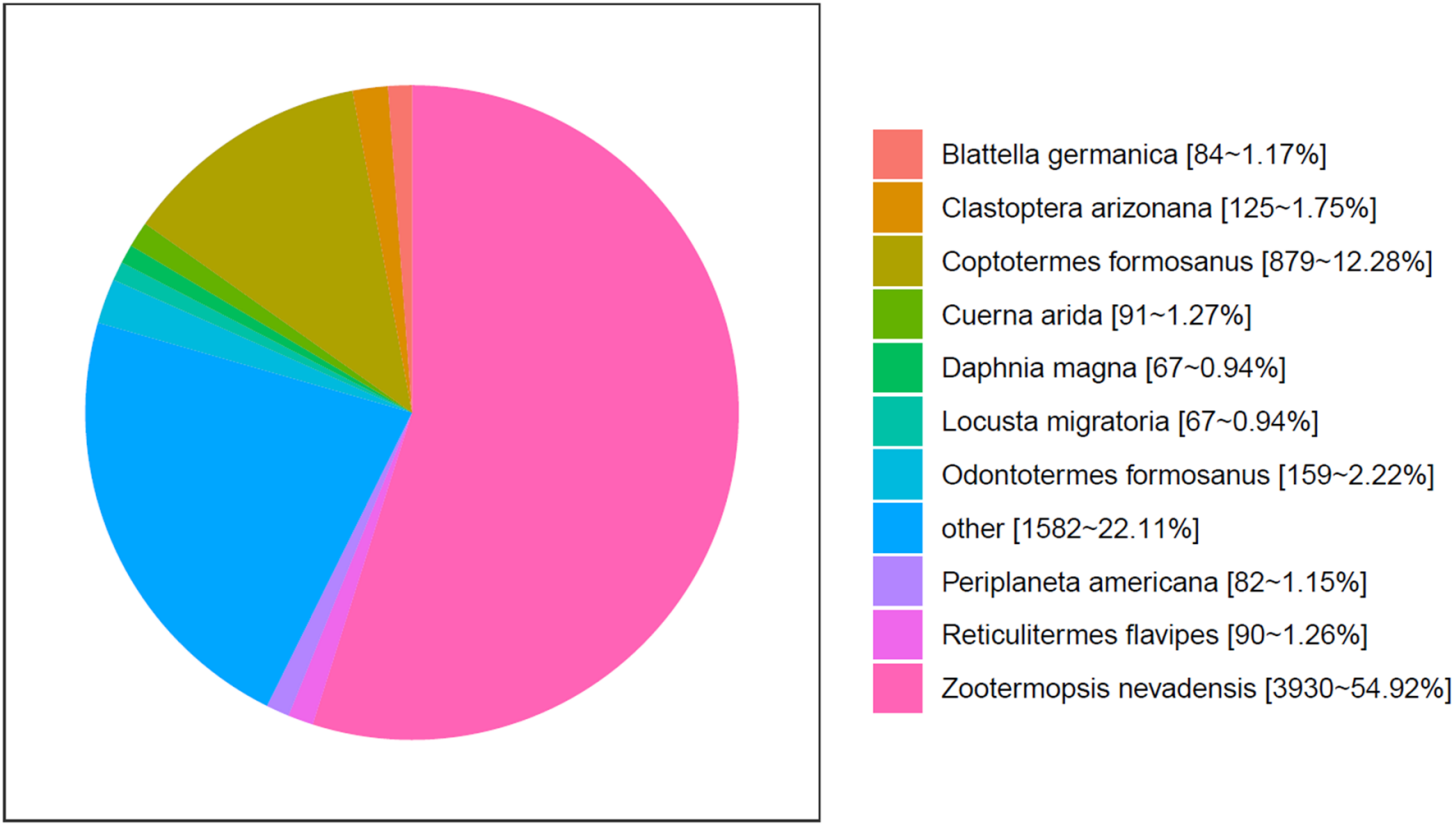
Nr classifications of all *O. formosanus* unigenes. Species distribution is shown as a percentage of the total homologous gene hits.

In total, 9888 transcripts were annotated in the database: 3062 were annotated in GO, 4299 in KEGG (KO), 3536 in KOG, 5856 in Swiss-Prot and 7156 in NR. Moreover, 2719 transcripts were not annotated in any of the eight databases. The highest unigene hit rates were clearly in insect genomes; *Zootermopsis nevadensis* (3930, 54.92%), other (1582, 22.11%), *Coptotermes formosanus* (879, 12.28%), *Odontotermes formosanus* (159, 2.22%), *Clastoptera arizonana* (125, 1.75%), *Cuerna arida* (91, 1.27%), *Reticulitermes flavipes* (90, 1.26%), *Blattella germanica* (84, 1.17%), *Periplaneta americana* (82, 1.15%), *Daphnia magna* (67, 0.94%) and *Locusta migratoria* (67, 0.94%) accounted for unigenes with Nr annotations (Fig. 1).

The GO database is maintained by the Gene Ontology Consortium and includes a clearly structured, clearly defined, universal and controlled vocabulary to describe the roles of genes and gene products in any organism. The GO annotation system is a directed acyclic graph that includes three categories: BP, MF, and CC.

Using Blast2GO software, the transcriptome of *O. formosanus* was successfully mapped to the three main functional processes, which comprised 51 GO terms.

**Figure 2:**
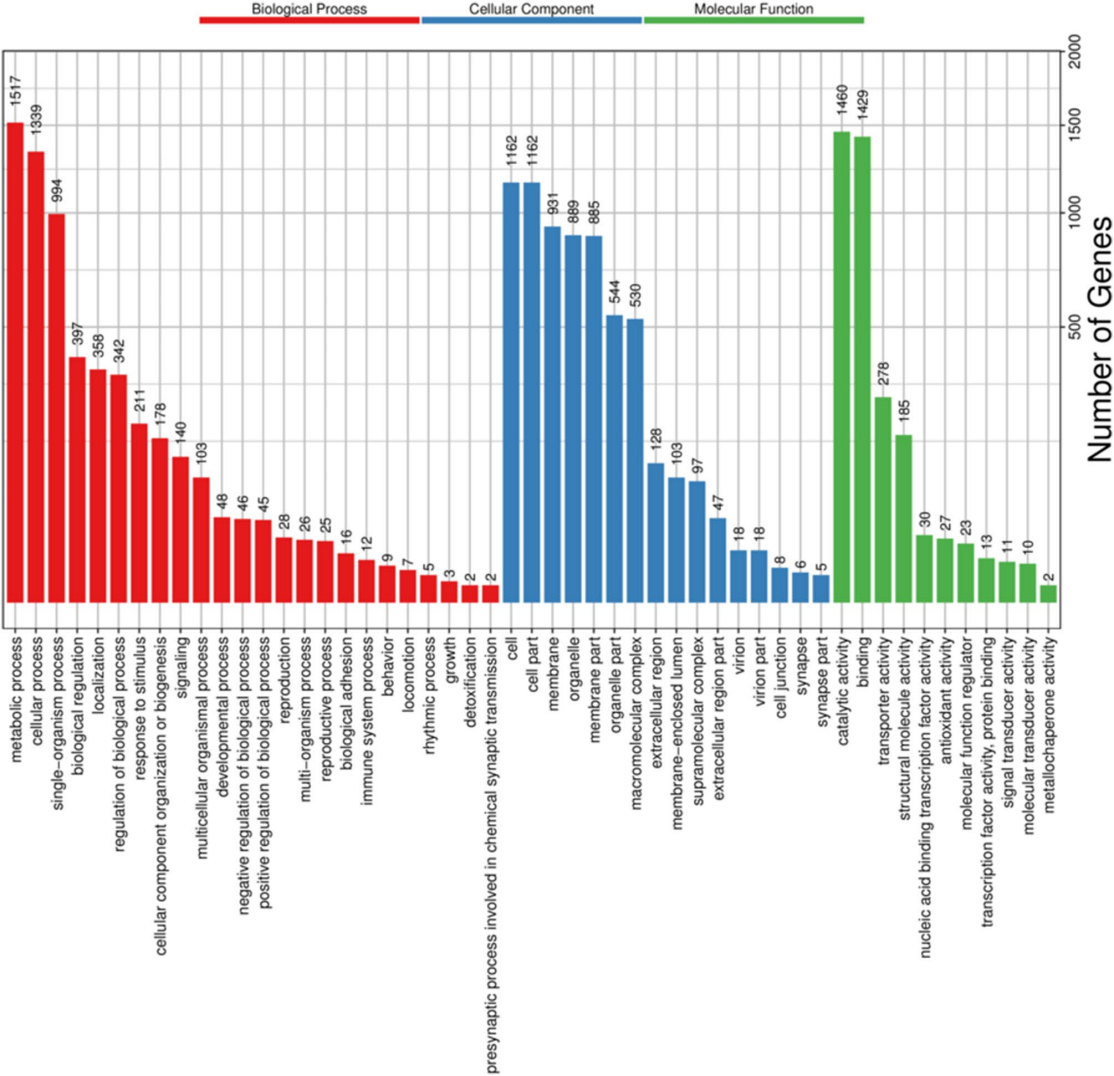
GO functional classifications of all *O. formosanus* unigenes. Red represents biological process; blue represents cellular component; and green represents molecular function. The x-axis represents GO categories; the y-axis (right) represents the number of transcripts.

**Figure 3:**
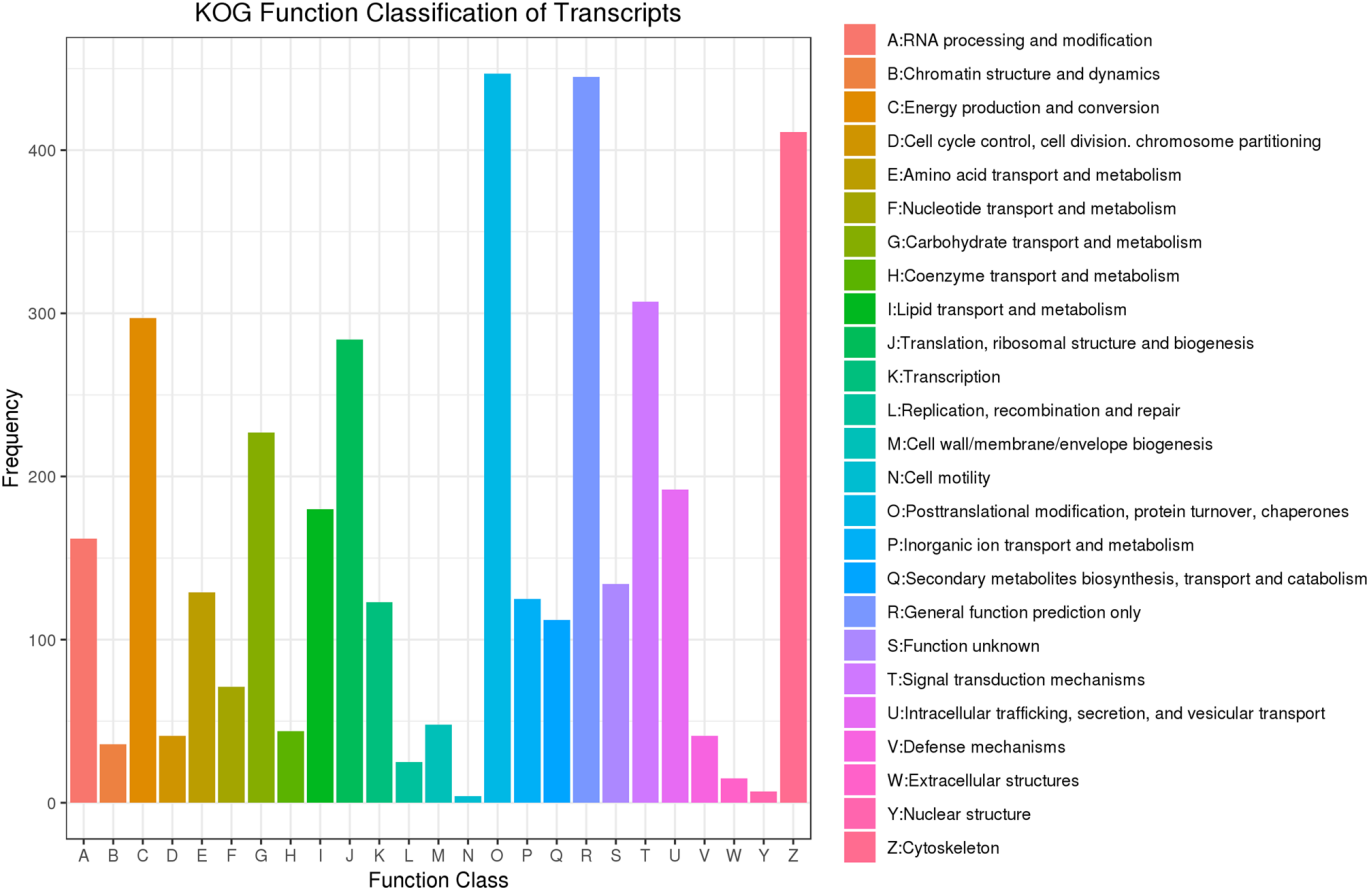
KOG annotations of putative proteins. The abscissa represents 25 KOG groups, and the ordinate shows the number of transcripts annotated to each group. The x-axis represents COG categories; the y-axis represents the number of transcripts.

The GO gene functional classification system classified 3062 unigenes into three main functional ontologies (BP, MF, CC) (Fig. 2). Based on the GO analysis, 2047 genes were classified as BP, 1831 genes were classified as associated with CC, and 2427 were associated with MF. With regard to the BP category, the main subcategories were metabolic process (1517) and cellular process (1339), followed by single-organism process (994). In terms of MF, catalytic activity (1460) and binding (1429) were the most highly represented. In the CC category, cell part (1162), cell (1162) and membrane (931) were highly enriched. In total, 3536 sequences had KOG classifications. They were divided into 25 KOG groups using Diamond with the following comparison parameters: − f 6 − e 1e − 2—more-sensitive, and the other parameters were set to the default values (Fig. 3). Among the 25 KOG classifications, the largest group was posttranslational modification, protein turnover, chaperones (447), followed by general function prediction only (445) and cytoskeleton (411). These results may be related to the fact that there is still little data on *O. formosanus* in the KOG database at this stage. A total of 6352 unigenes with unknown function were obtained by sequencing and are presumed to represent new genes specific to *O. formosanus*.

### DEGs in *O. formosanus *in response to *S. marcescens* infection

**Figure 4:**
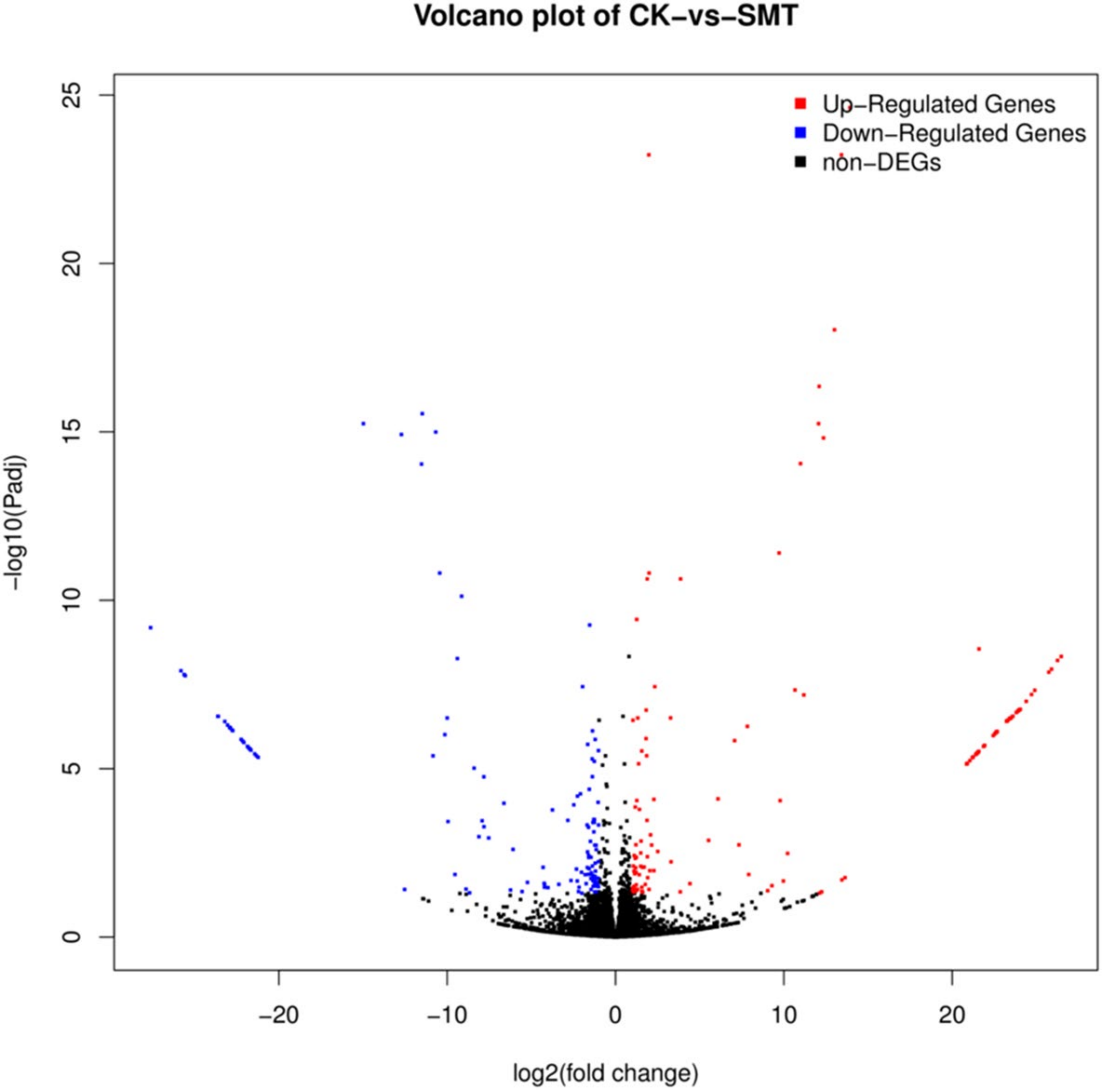
Comparison of gene expression levels between the CK library and SMT library. Red and blue spots represent significantly up-regulated and down-regulated genes, respectively. Black spots indicate no significant differences in gene expression.

**Figure 5:**
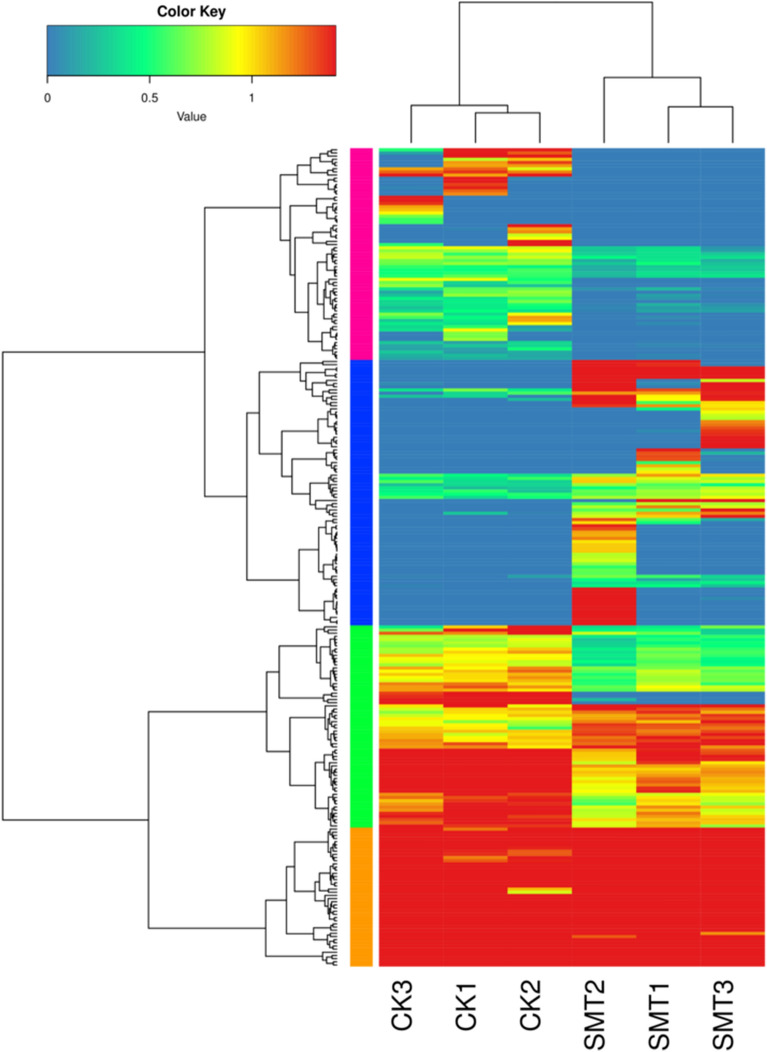
Overview of DEGs. Heatmaps illustrating differences in normalized log signal intensity for the identified *O. formosanus* genes. SMT1, SMT2 and SMT3 are *S. marcescens* treatment groups, and CK1, CK2 and CK3 are control groups. Red and blue indicate genes expressed at high and low levels, respectively. The colors from blue to red indicate gradually increasing expression.

To explore the mechanism of *S. marcescens* infection, we tried to identify DEGs that were up- or downregulated in *O. formosanus* infected with *S. marcescens* by using PacBio Sequel sequencing. To maximize the accuracy of expression measurement, data from three biological replicates were combined, FPKM values were computed using the combined data, and the results were compared between the replicate SMT and CK groups (Fig. 4). DEGs between the SMT and CK groups were considered significant when FDR < 0.5 and log_2_^FC^ > 1 or log_2_^FC^ < − 1. Among the 9888 unigenes, a total of 259 DEGs were identified (Fig. 5), of which 132 were upregulated and 127 were downregulated.

### DEG analysis

**Figure 6:**
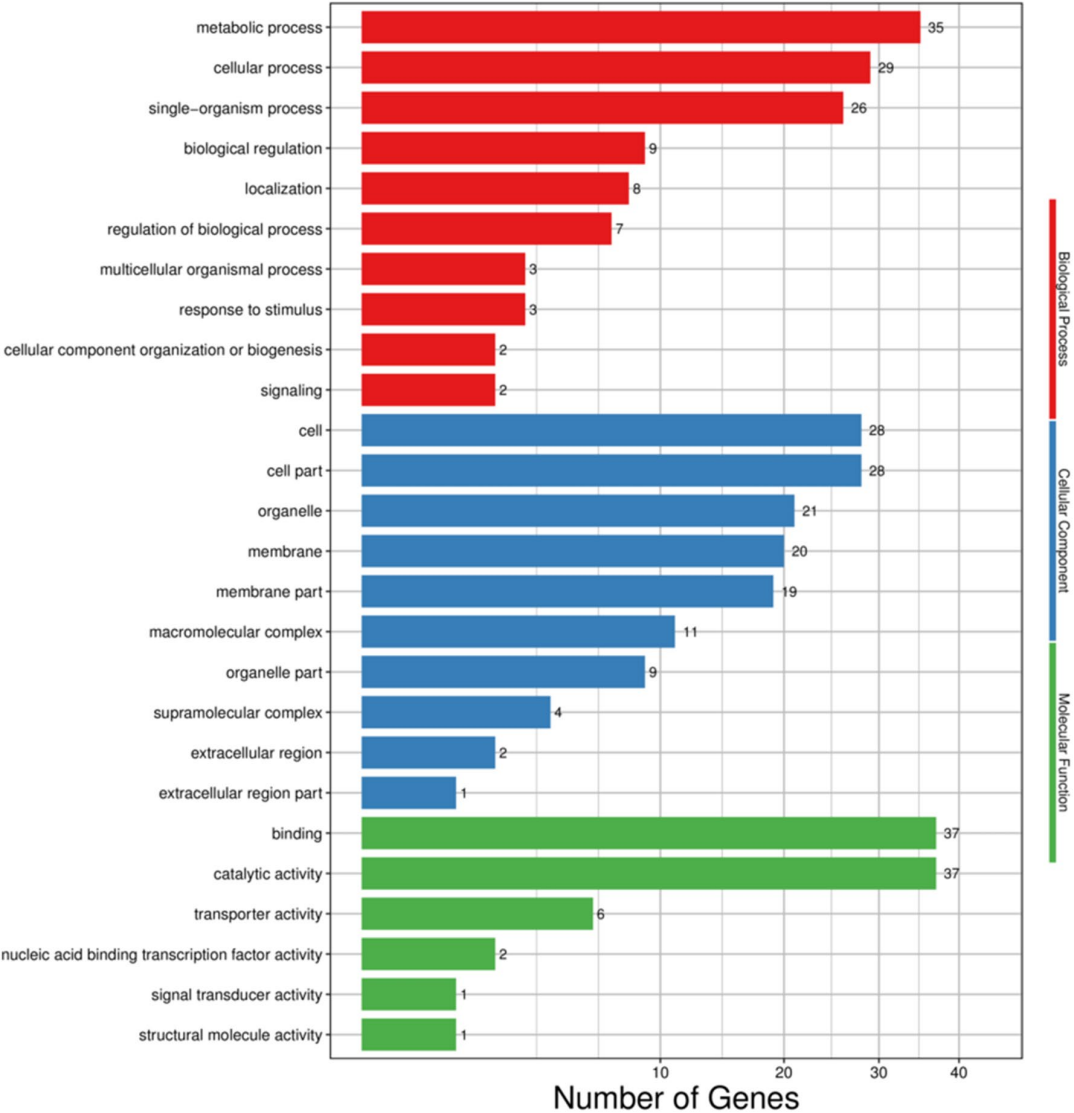
GO functional classification of DEGs in *O. formosanus*. Red represents biological process; blue represents cellular component; and green represents molecular function. The x-axis represents GO categories; the y-axis (right) represents the number of transcripts.

GO was used to classify the functions of the *O. formosanus* DEGs. A total of 69 unigenes were divided into 26 functional groups. For the BP category, the dominant subcategories were metabolic process (35) and cellular process (29), followed by single-organism process (26). In the CC category, cell part (28), cell (28) and organelle (21) were highly enriched. In MF, catalytic activity (37) and binding (37) were the most highly represented terms (Fig. 6).

We mapped the DEGs to the typical KEGG pathways and then determined the biological pathways involved in response to *S. marcescens* treatment. In total, 94 DEGs were assigned to 115 KEGG pathways (Supplementary Table S2). Pathways with p-values < 0.05 were considered highly enriched (Table 4). The DEG enrichment analyses showed that the top three pathways enriched in up- or downregulated genes in response to *S. marcescens* treatment were “Adrenergic signaling in cardiomyocytes” (12 unigenes), “Cardiac muscle contraction” (12 unigenes) and “AMPK signaling pathway” (6 unigenes). These annotations provide valuable resources for further study of the specific processes, functions and pathways of *O. formosanus* under *S. marcescens* treatment.

**Table 4:** Enriched KEGG pathways of DEGs in the *O. formosanus* transcriptome.

	KEGG pathway	DEGs with pathway annotation (gene number)	All genes with pathway annotation (background number)	P-value	Pathway ID
1	Adrenergic signaling in cardiomyocytes	12	181	0.000577	ko04261
2	Lipoic acid metabolism	2	3	0.001445	ko00785
3	AMPK signaling pathway	6	76	0.006427	ko04152
4	Cardiac muscle contraction	12	277	0.018618	ko04260
5	Glyoxylate and dicarboxylate metabolism	4	54	0.031064	ko00630
6	Other glycan degradation	2	13	0.032516	ko00511

## Discussion

Termites are among the most famous pests in the world, causing great damage, and their infestations are difficult to control. There have been some studies on termites using the molecular technology. Harrison et al. revealed molecular basis of termite eusociality using hemimetabolous genomes^30^; Korb et al. compared the genomes of *zootermopsis nevadensis* and *Macrotermes natalensis*, and focused on genes involved in communication, immune defenses, mating biology and symbiosis that were likely important in termite social evolution^31^; Poulsen et al. reported the complementary symbiont contributioned to plant composition in a fungus-farming *Macrotermes natalensis* worker using the high-quality annotated draft genomes^32^; Terrapon et al. studied the molecular traces of alternative social organization in a termite genome by sequencing the genome and stage specific transcripts of *Zootermopsis nevadensis* (Blattodea) and comparing them with similar data of eusocial Hymenoptera^33^; Wu et al. clarified that soldier-biased gene expression in *Reticulitermes flavipes* implied functional specialization of the defensive caste using RNA sequencing^34^. However, few studies on the molecular biology of termites by ‘third-generation’ sequencing technology, such as the excavation, cloning and functional identification of genes, are available. Although, we have proved the insecticidal activity of *S. marcescens* through some bioassay experiments, but the specific insecticidal mechanisms of *S. marcescens* on *O. formosanus* are unclear, and the immune responses of *O. formosanus* to *S. marcescens* have not been clarified. Therefore, we use the third-generation high-throughput sequencing technology to explore these issues. In this study, third-generation SMRT sequencing technology was used to sequence for the first time the full-length transcriptome of termites under *S. marcescens* stress, and 10,364 full-length unigenes were obtained. In addition, 9888 unigenes were successfully annotated without a reference genome. These results indicate that the sequencing method used in this study yields rich data with high quality. These results will be helpful to clarify the action mechanisms of *S. marcescens* on *O. formosanus,* and explore the relationship between *O. formosanus* and *S. marcescens*. In addition, this study will identify the immune response of *O. formosanus* to *S. marcescens*, which will provide a theoretical foundation for the development of new immunosuppressants for *O. formosanus*. Furthermore, these high-quality unigene data will provide an important reference for subsequent functional genomics research, including termite-related gene discovery, cloning and functional identification.

### Oxidative stress is associated with *S. marcescens* in *O. formosanus*

In vertebrates, ferritin is mainly present in the cytoplasm. Ferritin includes both heavy (H) chain and light (L) chain subunits^35^. When the content of free iron in cells is insufficient, ferritin can release stored iron ions and maintain normal life activities; when too many free iron ions are present in cells, large amounts of ferritin can be synthesized to store the excess iron ions. Thus, ferritin can prevent the damage to lipids and proteins caused by Fenton reactions and participate in the process of eliminating antioxidant stress^36^. As in vertebrates, ferritin in insects can also reduce antioxidant stress, acting as a protective agent against cytotoxicity. Insect ferritin is also composed of two subunits, heavy chain homolog (HCH) and light chain homolog (LCH) subunits^37^. These two subunits of ferritin are assembled depending on the ingestion, storage and release of iron ions in the body to form heteropolymers in different proportions to achieve the purpose of regulating iron ions. Overall, ferritin plays an important role in the various stages of organism proliferation, growth and development.

The most basic functions of ferritin in the body are to regulate iron metabolism and maintain iron homeostasis. It has been reported that ferritin in *Drosophila* played an important role in combating oxidative stress. When iron ions were added to the diet of *Drosophila*, the expression of the LCH and HCH subunits was significantly upregulated^38,39^. Similarly, ferritin played a role in regulating oxidative stress in mosquitoes^40^. In all animals, the expression of the LCH and HCH genes can be induced by dried blood^40^. As an iron chelator, ferritin can eliminate the Fenton reactions caused by excessive iron ions in insect food, thus protecting the body from reactive oxygen species (ROS). During the attack by invading pathogenic microorganisms, ferritin can store excess iron ions in the body, thereby inhibiting the growth of the pathogens. A previous study showed that the content of ferritin in the hemolymph increased significantly when beetle larvae were stimulated by fungi^41^, and another study showed that the content of ferritin in bumblebees increased significantly after iron stimulation^42^, and it has been reported that after LPS or bacterial stimulation, the expression of ferritin mRNA in horseshoe crabs was upregulated^43^. At present, the cause of the change in ferritin in *O. formosanus* under oxidative stress and how ferritin in *O. formosanus* helps to resist oxidative stress have not been reported. In our study, we found that the expression of ferritin was downregulated, which may be related to bacterial infection. This result might be due to a large quantity of iron ions obtaining from the host after bacterial infection, resulting in a decrease in the expression of ferritin.

Insect transferrin (TF) can be used as iron transporter, antibiotic or larval hormone regulatory protein to perform physiological functions. TF plays an important role in insect resistance to oxidative stress. It has been found that heat treatment, fungal invasion and H_2_O_2_ stimulation can lead to upregulated TF gene expression in the fat body of beetles and subsequently to the upregulation of TF gene expression in the hemolymph^43^. To prevent the harmful effects of iron, extracellular iron binds to TF and protects tissues from iron-induced oxidative stress damage^44^. In addition, insect TF plays an important role in fighting against bacterial infection^45,46^. Toe et al. stimulated *Macrobrachium rosenbergii* with *Aeromonas hydrophila* and confirmed that the content of TF in *M. rosenbergii* changed significantly^47^. In the present study, our results showed that the expression of TF in termites changed significantly after bacterial infection, and five insect TF genes were found to be downregulated (1.9–12.7-fold), while one gene was upregulated (23.31-fold), in *O. formosanus* following *S. marcescens* infection. The expression of the most TF genes was downregulated, which would lead to the reduction of transferrin synthesis, so that the excessive iron could not be eliminated in time, and further lead to the decline of antioxidant stress capacity of termites. This might be one of the reasons for the high insecticidal activity of *S. marcescens* on *O. formosanus*, which needed further experimental verification.

### The MAPK pathway is associated with bacterial stress in *O. formosanus*

The mitogen-activated protein kinase (MAPK) pathway is an important protein-based signaling system in which eukaryotic cells transduce extracellular signals into cells to cause cellular responses^48^. An extensive and in-depth study of apoptosis-related genes and apoptosis signaling pathways found that the MAPK pathway plays an important role in regulating bacteria-induced apoptosis.

In vitro experiments have identified a large number of bacteria as mediators of apoptosis. These bacteria include Gram-positive bacteria, Gram-negative bacteria, and mycobacteria. Tseng et al.^49^ showed that the MAPK pathway was the main pathway in AGS apoptosis induced by *Helicobacter pylori*-derived Smase (Sphingomyelinase). Watanabe et al.^50^ used *Porphyromonas gingivalis* to infect human gingival epithelial cells (GECs), and the results showed that *P. gingivalis* acted selectively on the MAPK pathway. Dahan et al.^51^ used enterohemorrhagic *Escherichia coli* (EHEC) to infect intestinal epithelial cells (T84) for 3 h and showed the phosphorylation of three protein kinases (ERK1/2, P38 and JNK) in the MAPK family, demonstrating the role of the MAPK pathway in EHEC infection. These studies showed that the MAPK pathway played an important role in signal transduction related to bacterial-induced apoptosis. In our reseach, 14-3-3 protein bet/alpha (YWHAB) in the MAPK pathway was strongly downregulated (tenfold), meanwhile, YWHAB also existed in a immune pathway, JAN/STAT signaling pathway which was the branch of P13K-Akt signaling pathway in *O. formosanus*. This suggested that certain immune function of *O. formosanus* was inhibited to some extent by *S. marcescens* infection. This might be one of the reasons for the high insecticidal activity of *S. marcescens*.

MAPKs also play a role in phagocytosis and the survival of some microbial pathogens in macrophages. The process by which bacteria invade cells is often associated with activation of the MAPK pathway. For example, MAPKs in macrophages can be activated by bacterial lipopolysaccharide (LPS). Infection of epithelial cells with pathogenic *Staphylococcus aureus* can induce the activation of genes involved in the MAPK pathway (ERK1/2, P38 and JNK)^52^. In contrast, *Yersinia pestis* can reduce the activity of genes involved in the MAPK pathway (ERK1/2, P38 and JNK) in both macrophages and epithelial cells. In addition, curcumin has been found to inhibit the expression of macrophages by inhibiting MAPK/NF-κB signaling pathway^53^. This indirectly indicates that MAPK pathway is associated with phagocytosis. In our study, following *S. marcescens* infection, three genes involved in phagocytosis were upregulated (1–23-fold), and one gene was downregulated (22-fold). This phenomenon indicated that phagocytosis in termites was active but limited in the process of resistance to *S. marcescens* infection. Therefore, the MAPK pathway in *O. formosanus* may mediate *S. marcescens*-induced stress responses. However, further studies are needed to establish the possible regulatory role of the *O. formosanus* MAPK pathway during infection by *S. marcescens*. Exploring the relationship between *O. formosanus* and *S. marcescens* will help to elucidate the mechanisms of *S. marcescens* at the molecular level and provide an important theoretical basis for the development of new immunosuppressants for *O. formosanus*.

### The AMPK pathway is associated with bacterial stress in *O. formosanus*

AMP-activated protein kinase (AMPK) is a serine/threonine kinase. It is a key metabolic and stress sensor/effector that is activated under conditions of nutrient deprivation, strenuous exercise or heat shock^54^, as well as an important regulator of cellular energy balance^55^.

The energy state of an organism is closely related to its rate of aging, and a low energy state delays aging in multiple species^56^. AMPK regulates senescence-associated pathways, including TOR/S6K, FOXO, sirtuins, cAMP response element binding protein (CREB)-regulated transcriptional coactivator (CRTCs), NF-κB signaling and other mediators^57^. Studies have shown that activation of AMPK is associated with prolonged life and improved health^55^. Funakoshi et al.^58^ showed that in fruit flies, the AMPK pathway could be activated by dietary restriction, which could extend the lifespan of fruit flies. Su et al.^59^ found that feeding alpha-ketoglutarate could activate AMPK signaling and prolong the lifespan of fruit flies. Piegholdt et al.^60^, by feeding prunetin, found that the prolongation of the lifespan of male fruit flies was dependent on the changes in energy homeostasis regulated by AMPK. Yang et al.^61^ found that dietary administration of β-guanidinopropionic acid at a concentration higher than 900 mmol led to a significant extension of the lifespan of *Drosophila melanogaster* in repeated experiments. Ulgherait et al.^62^ found that when the AMPK pathway was activated, cells cleared their aging and damaged components through autophagy. Furthermore, the lifespan of fruit flies was prolonged when the amount of AMPK in the intestine was increased. Burkewitz et al.^57^ showed that AMPK could prolong fruit fly lifespan by inhibiting the activity of the CRTC-1 protein. When AMPK content increased, CRTC-1 could not bind to CREB, thus ensuring the normal function of mitochondria throughout the body and prolonging life. Funakoshi et al.^58^ found that overexpression of lkb1 increased the level of phosphorylated AMPK, leading to prolong the fruit fly life. At present, studies of the AMPK pathway and related genes have not been reported in termites. In our study, following *S. marcescens* infection, three genes in the AMPK pathway were upregulated (1.15–23.28-fold), and three genes were downregulated (1.27–22.76-fold). These results indicate that the AMPK pathway in *O. formosanus* may mediate *S. marcescens*-induced stress responses. Other studies have found that the activation of AMPK pathway was closely related to energy metabolism. When insects were stimulated (pesticides, starvation, etc.), AMPK pathway would be activated to reduce the anabolism of cells, reduce the consumption of materials and energy, so as to maintain the survival of cells. It could also activate autophagy to resist the invasion of some intracellular pathogens. In our results, we found that there were significant changes in the expression of a large number of genes in this pathway, which may be related to the resistance of *O. formosanus* to *S. marcescens* invasion. Termites control energy metabolism by activating AMPK, reduce the loss, and use a lot of energy to resist *S. marcescens* invasion. However, further studies are needed to establish the possible regulatory role of the AMPK pathway in *O. formosanus* infection by *S. marcescens*.

## Supplementary information

Supplementary Table S1.

Supplementary Table S2.
